# Multi-Person Localization Based on a Thermopile Array Sensor with Machine Learning and a Generative Data Model

**DOI:** 10.3390/s25020419

**Published:** 2025-01-12

**Authors:** Stefan Klir, Julian Lerch, Simon Benkner, Tran Quoc Khanh

**Affiliations:** Laboratory of Adaptive Lighting Systems and Visual Processing, Technical University of Darmstadt, Hochschulstr. 4a, 64289 Darmstadt, Germany; lerch@lichttechnik.tu-darmstadt.de (J.L.); benkner@lichttechnik.tu-darmstadt.de (S.B.); khanh@lichttechnik.tu-darmstadt.de (T.Q.K.)

**Keywords:** people localization, thermopile array, multi-person detection, infrared array sensor, generative IR data

## Abstract

Thermopile sensor arrays provide a sufficient counterbalance between person detection and localization while preserving privacy through low resolution. The latter is especially important in the context of smart building automation applications. Current research has shown that there are two machine learning-based algorithms that are particularly prominent for general object detection: You Only Look Once (YOLOv5) and Detection Transformer (DETR). Over the course of this paper, both algorithms are adapted to localize people in 32 × 32-pixel thermal array images. The drawbacks in precision due to the sparse amount of labeled data were counteracted with a novel generative image generator (IIG). This generator creates synthetic thermal frames from the sparse amount of available labeled data. Multiple robustness tests were performed during the evaluation process to determine the overall usability of the aforementioned algorithms as well as the advantage of the image generator. Both algorithms provide a high mean average precision (mAP) exceeding 98%. They also prove to be robust against disturbances of warm air streams, sun radiation, the replacement of the sensor with an equal type sensor, new persons, cold objects, movements along the image frame border and people standing still. However, the precision decreases for persons wearing thick layers of clothes, such as winter clothing, or in scenarios where the number of present persons exceeds the number of persons the algorithm was trained on. In summary, both algorithms are suitable for detection and localization purposes, although YOLOv5m has the advantage in real-time image processing capabilities, accompanied by a smaller model size and slightly higher precision.

## 1. Introduction

The presence detection of a person with and without movement is essential within many aspects of building automation, such as smart lighting systems [1], elderly fall detection [2,3,4] or movement tracking [5,6]. A thermopile sensor array uses a grid of thermopiles to record a low-resolution infrared (IR) image representing a 2D thermal landscape of the area within the array’s field of view (FOV) [7]. The thermal landscape texture is formed by the interaction of IR sources, such as occupants or heaters, with the IR absorption and/or reflection characteristics of surrounding surfaces. Based on the difference in two time-consecutive images, properties like the number of occupants or their direction of movement can be detected and localized [5,8,9]. In addition, such thermopile arrays preserve the occupant’s privacy due to their low resolution compared to other image based systems like optical cameras [10,11,12].

By leveraging the privacy advantage of a thermopile array in previous discussed applications such as smart lighting systems, elderly fall detection or movement tracking, only anonymized data are recorded. At the same time, valuable information can be obtained about the number of occupants and their speed and direction of movement. For these applications, no explicit information about the exact person, gender or age of the people in the FOV are required. In industrial and office environments, such as meeting rooms with high security and privacy standards, optical RGB or monochrome cameras can therefore not be used. Based on this challenge and new potential fields of application with low resolution, privacy-preserving thermopile sensors, the following research aims to utilize state-of-the-art algorithms that achieve two goals: (1) to robustly distinguish between a person and a dynamic heat radiation object and (2) to conceive an algorithm that is also robust to changing environments like different installation locations.

## 2. Related Work

In the context of this paper, we further distinguish between non-disturbed and disturbed environments. Non-disturbed environments are defined as environments without any other infrared/heat-radiating object than the detection objects, in this case humans. This is in contrast to disturbed environments where people and additional heat-radiating objects are located, like a computer, a hot cup of coffee or a heater. The following literature review discusses statistical methods of person detection. Subsequently, data-driven approaches are examined and divided into two categories: two-stage and single-stage detectors. The main focus of the literature review is to indicate robust algorithms that can distinguish between humans and other heat-radiating objects with a high accuracy based on a minor set of example data with an execution time less than the frame rate of an IR array sensor. These goals are specified to create a fundamental system to extract humans, with respect to privacy due to the low resolution of IR images, for further processing.

### 2.1. Object Detection in Infrared Images

Localization enables the tracking of peoples’ movement [5,8] and the ascertainment of a human’s facing direction with a maximum accuracy of 95.1% [9]. Simple threshold algorithms are able to detect and follow the movement of a human within the detection space in non-disturbed environments with a 1 × 8-pixel array sensor [13]. Further research focused on algorithms that filter the sensors’ output, divide the background from human occupants and use a dynamic threshold [5,8,14,15] or a probability function [16]. As the basis of the aforementioned research, a single-pixel sensor or pixel arrays of the size 1 × 8, 8 × 8 or 24 × 32 pixel have been examined. These detection approaches can be incorporated into edge computing solutions due to their low to moderate computational complexity whilst providing higher processing speed as the frame rate of the sensor [14]. As a disadvantage, they can poorly handle disturbed environments, since they assume that higher temperature areas are humans. Likewise, a higher air temperature in the environment will lead to a smaller signal-to-noise ratio, resulting in a degraded accuracy [14,17].

Data-driven deep learning techniques for person detection often apply a convolutional neuronal network (CNN) combined with layers that incorporate time like long short-term memory (LSTM) [2,4,6], recurrent neuronal network (RNN) [4] or a gated recurrent unit (GRU) [2,4]. Likewise, support vector machines (SVM) [9] are implemented as classifiers with a CNN for feature extraction. These data-driven approaches can be specialized for certain tasks, environments and disturbing objects with a ground truth of data. Data quality and the amount of labeled samples to calculate a loss during training are a key factor for high accuracy and stable results in changing environments. For fall detection, for example, the precision differed for a single LSTM Layer from 85% in the case of perpendicular falls to 100% for parallel falls for a 8 × 8-pixel sensor with 300 training samples [2]. Vandersteegen et al. [18] proposed, in 2022, an ultra-lightweight CNN-based detector inspired by YOLOv2t that runs with full frame speed on a microcontroller and achieved an accuracy of 79.9%. This algorithm requires a ground truth image without people and therefore exhibits inaccuracies in dynamic hot disturbing objects. A similar behaviour was observed by Chen et al. [9] with a sparse training dataset of 250 images; an accuracy of 95.1% was achieved [9].

These investigated papers point out that object detection approaches can achieve high detection accuracy in low-resolution thermal images. However, real-world scenarios present disturbed complex environments. Such application scenarios are within the scope of current research interest. For this reason, the literature is reviewed for suitable and state-of-the-art object detection algorithms to further increase detection robustness with regard to environmental disturbances in the sensors’ FOV.

Current object detection algorithms can be divided in single-stage and two-stage algorithms. Both categories will be examined in more detail in the next sections.

### 2.2. Two-Stage Detectors

Two-stage algorithms separate the localization task from the classification task. They can be a combination of, e.g., a conventional segmentation method like selective search [19] combined with a convolutional neural network (CNN). Hence, the algorithm first generates region proposals (first stage) and then classifies them (second stage) [20]. This method was introduced by Girshick in 2014 [21] and has been continuously developed since.

On this foundation the two-stage region-based CNN (R-CNN) algorithms family was introduced by Wu et al. [21]. In the first stage, category-dependent region suggestions are defined. Further, in the second stage, features are extracted by a CNN, based on these regions, and category specific labels are generated with, e.g., an SVM [22] algorithm [23]. This approach divides the detection problem though the class independent region suggestions into two independently addressable localization and classification problems. One downside is that algorithms in both stages must be trained individually and the optimization of the full pipeline is therefore of higher complexity [24]. Multiple improvements based on the R-CNN approach have been conducted in subsequent years [25,26,27,28]. Currently, the Mask R-CNN algorithm by He et al. [29] provides the highest average precision of the R-CNN family. In particular, it implements a simultaneous calculation of the segmentation masks and bounding boxes.

A common characteristic of the aforementioned algorithms is a high computationally complexity during their training phase that consequently results in a high precision. Especially in person localization tasks, the topics for data evaluation in edge computing applications, which exhibit limited memory and computing capacities as well as algorithms with short inference time, are gaining more research interest.

### 2.3. Single-Stage Detectors

In 2016, J. Redmon suggested [30] a single-stage detector You Only Look Once—YOLO—based on a single neuronal net. YOLO, unlike R-CNN, considers object detection not as a classification problem, but as a regression problem from image pixels to spatially separated bounding boxes and associated class probabilities.

The main drawbacks of YOLO are that the network has problems with small objects appearing in groups as well as detecting objects with new or unusual aspect ratios [30].

Since its initial release in 2016, YOLO has undergone continuous development and is now available in numerous updated versions. A YOLO develops futher, it reaches a better accuracy, faster speed and the ability to detect a greater amount of various objects. YOLOv5 was the first implementation in Python and is based on PyTorch, whereas the previous implementations are in PASCAL. A key advantage of YOLO is the real-time ability to achieve frame rates over 30 fps with 1280 × 1280 pixel images [31].

Another recent research branch deploys transformers for the purpose of object detection. Transformers include attention mechanisms to gather information of the entire input sequence and have the advantage of a global view that results in an optimized memory in the pipeline. In 2020, the Detection Transformer (DETR) [32] was introduced, which implements a transformer encoder–decoder head to generate a set of box predictions based on image features from an CNN head. DETR is also a single-stage detector and has the same precision on the MS COCO dataset [33] as the two-stage Faster R-CNN [27], with half the computational power [32]. Furthermore, the algorithm carries out all detections in parallel, and in the comparison, the model runs at 28 fps while using 118,000 training data with larger images than 480 × 1333 pixel. Another 2021-introduced approach is the Swin Transformer [34] which has the highest accuracy of all mentioned algorithms on the MS COCO dataset [24]. This transformer splits the input image in multiple non-overlaping patches and post-processes the data after a linear embedding layer with multiple modified self-attention computation blocks in four stages. The complexity increases linearly with the input images’ size [34].

In summary, the two-stage approaches—R-CNN family—were designed to be more precise than the single-stage algorithms. The new versions of YOLO and the recent research in transformer algorithms show that single-stage algorithms are now similar or even more precise than their two-stage counterparts while providing a higher execution speed for real-time image processing. Furthermore, during the work on this paper, new approaches were released, which show that transformer-based detection algorithms are of great research interest [35].

### 2.4. Issues in Current Object Detection for IR Images

Current object detection algorithms are focused on RGB camera images with a higher resolution rather than the infrared array images that are monochromatic with a low resolution of 4 × 4 pixel that can reach up to 32 × 32 pixel [36]. In research by Anyanwu et al. [36], a single- and a two-stage detector algorithm is applied to archive high accuracy, robustness and speed in occupancy detection in IR images with multiple persons and heat-radiating disturbance objects. In 2021, Petrova et al. [12] published a review paper of object detection algorithms based on IR images. These studies assume a non-disturbed environment and the detection of only a single person in the sensors’ FOV [10,11,37,38,39]. However, this current paper includes multiple persons in real disturbed environments with the possibility to switch to an untrained environment and still maintain accuracy. In addition, a generative model to construct annotated infrared images with labeled occupancy locations based on a few manually pre-labeled data is introduced. Due to current research, single-stage detectors have a similar accuracy and higher computation speed than two-stage detectors; the focus of this publication is on single-stage detectors. During the code implementation phase, YOLOv5 was the latest stable YOLO release. Han et al. [40] showed in their 2022 survey about vision transformers that DETR is a good choice for object detection and this will therefore be analyzed for infrared array images. These two single-stage algorithms, YOLOv5 and DETR, will be further evaluated during the course of this study.

Based on these algorithms, the aim of this paper is to localize multiple human occupants within low-resolution 32 × 32-pixel infrared images with the the sensor output’s maximum speed, thus preserving their privacy. Another major goal is to achieve robustness against changing environments as well as environmental disturbances like different rooms or non-occupant heat radiation sources, e.g., laptops, hot cups, air flow or heating.

## 3. Experiment

This section highlights the experimental setup for collecting IR image data, a generative labeling and data augmentation model and the training procedure.

### 3.1. Data Collection

A commercially available sensor with a 32 × 32-pixel resolution is utilized. This sensor offers a good balance between a low enough resolution to preserve privacy and simultaneously a sufficiently high resolution to distinguish multiple objects and persons in the field of view (FOV). The sensor model HTPA32 × 32dR2L1.9/0.8HiC[CH] manufactured by Heimann Sensor GmbH was arbitrarily selected for data capturing. This sensor series is advertised by Heimann Sensor GmbH (Eltville am Rhein, Germany) as being designed for person detection. The specifications are illustrated in Table 1.

**Figure 1:**
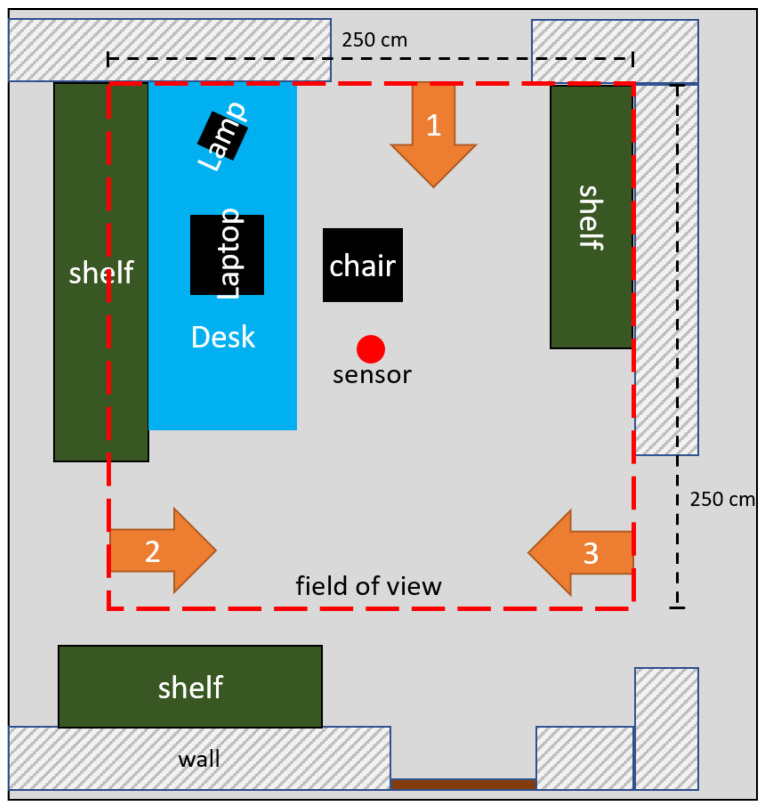
Schematic structure of the indoor office-like test environment with marked access points: 1, 2 and 3. The red dot marks the position of the thermopile sensor and the red dashed lines frame the field of view of the sensor, which equal 2.5 m × 2.5 m (7.8 cm × 7.8 cm pixel size on the floor). The sensor was installed at a height of 2.3 m on the ceiling, which measured heights up to 2.4 m.

After a literature search for labeled thermal images, no dataset with the following properties could be found: annotated multiple persons, disturbing objects with a resolution equal or greater than 32 × 32 pixel and an overhead installation position. Therefore, a suitable training dataset had to be created for this paper. For data collection, the environment is displayed in Figure 1 with three possible entries/exits as well as the sensor position (red dot) and field of view (red dashed rectangle). This scenario mimics an indoor office context, with one workplace and an overhead view of the infrared array.

According to the literature recommendations, the following parameters were chosen for generating a diversified dataset: occupants [41], clothing [3,42] and poses (standing, sitting, small movements, walking) [41]. In total, three scenarios were carried out by four male participants. The characteristics of each male person regarding height and hair type is collected in Table 2.

The four participants wore either spring, summer, autumn or winter clothing for data collection. The following actions were performed to gather the data:

1. Each person individually walked into the room, took a place at the working desk, typed something on the laptop, stood up and left through another exit. Each person accomplished this scenario six times with six different routes, resulting in about 1800 captured images.
2. Two people entered from different entrances, passing each other and leaving the field of view (FoV) at different exits. In total, 920 images were taken.
3. The area within the FoV was completely cleared of all objects and individual images of the participants were taken. Each person was captured standing, walking and sitting centrally under the sensor and in the four corners, as well as on the four borders of the test environment. The test subjects were encouraged to perform a variety of poses, such as sitting with legs stretched out, walking with long steps, or standing with their arms crossed. This scenario yielded roughly 350 images.

As a result, 3071 real-world images were collected and manually labeled. Of these images, 2150 (70%) were utilized as training data. Out of the remaining images, 614 (20%) were utilized as validation data and 307 (10%) as test data. Random selection determined the precise assignments.

### 3.2. Generative Data Creation Model

Data-driven algorithms, such as YOLOv5 and DETR, usually require a larger number of labeled data to perform in a robust and accurate manner. Since data collection and labeling is a highly time-consuming process, a novel generative model for an automatic creation of labeled infrared image data has been implemented. The infrared image generator’s (IIG) aim is to generate realistic images with automatic labeled persons and heat-radiating objects based on a small set of pre-captured and pre-labeled real-world data. By passing parameters like the number of occupants and interfering IR sources to the IIG, it generates an entirely new IR image with a correct scene and IR source(s) annotation. The image background and object temperature can be varied individually. Images created this way are then further augmented by methods like image rotation or flipping. Combining real-world data collection, IR image construction with the proposed IIG and additional augmentation yields a sufficient dataset for data-driven algorithms like YOLOv5 or DETR.

Creating an image with the IIG follows three steps: generate a background, place a specified number of occupants and finally add noise or interfering thermal sources to the image. Each of these three phases are explained in greater detail subsequently:

- Background generation: The background is modeled as a stretched normal distribution. By determining the function parameters, arbitrary backgrounds can be created.
- Inserting occupants: Crop occupants from labeled images and place a predefined number of persons in the image.
- Noise and interfering thermal sources: Thermal sources for noise are added to the image. Two kinds of noise are added: (1) As stretched normal distributions or (2) as cropped image fragments from the labeled data. Finally, the border of the inserted occupant image parts as well as the noise fragments from labeled data are blurred with a Gaussian filter to fit into the new background.

**Figure 2:**
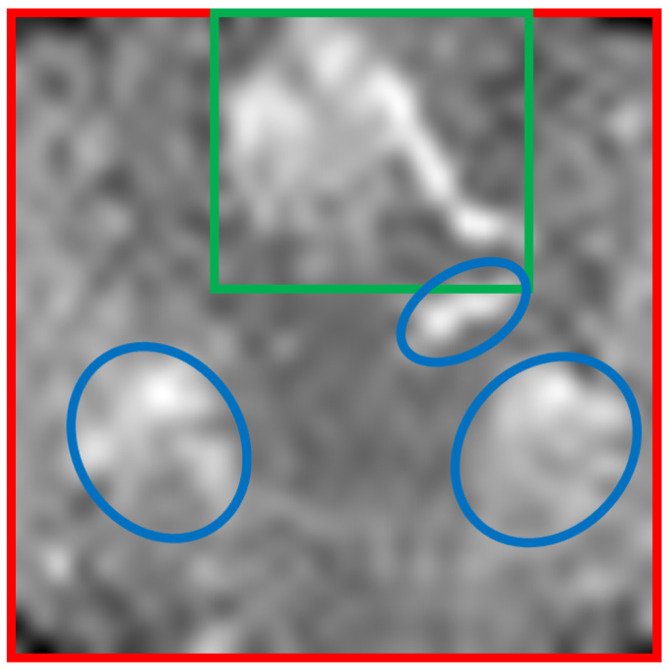
Real example 32 × 32 px image of the infrared array with a highlighted person (green box), disturbances (blue circles) and background (red box).

Figure 2 visualizes these parts in a real image.

The three parts—background, person and disturbances—were further analyzed with temperature histograms. The background is similar to a stretched normal distribution. The obtained temperature distributions of the humans were of high complexity and very diverse. Heat-radiating disturbances could be separated into two groups: (1) disturbances that are similar to a hot spot that have a stretched normal distribution and (2) a randomly distributed appearing second group. With this in mind, backgrounds and some disturbances are mathematically modeled, while humans and complex disturbances are extracted from real images.

**Figure 3:**
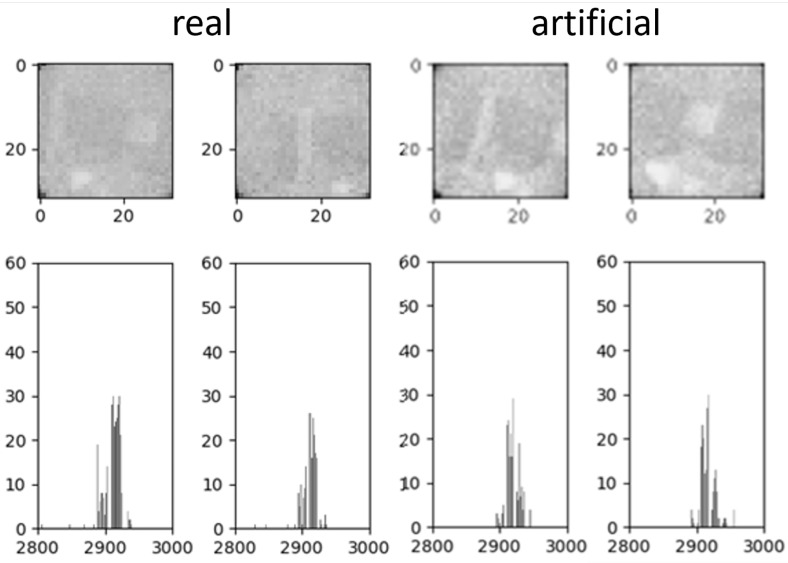
Comparison of two randomly selected real measured images on the left side and two artificial backgrounds on the right side. The real and generated images have a similar representation in the histogram.

As a base dataset for the IIG algorithm, a total of 3400 images, divided into 2000 images with people and disturbances and 1400 with only background and minor disturbances of the environment, were used. The 2000 images are equivalent to the training dataset from Section 3.1. However, in this dataset, only images were included in which a person is completely visible in the FoV of the sensor. Therefore, images in which a person exits and enters the FoV were excluded to work only with full representations of people. All 1400 background images were newly captured and are only used for a kernel density estimation (KDE) with an optimization of the bandwidth to fit the given backgrounds generally. This led to a non-parametric generative model for the backgrounds, where 32 × 32-pixel images can be drawn from to create new backgrounds. Figure 3 shows two real measured images and two artificial images. For the next step, the backgrounds were set to a random temperature between 18 °C and 25 °C, which corresponds to a common office temperature, by adding a temperature delta between the average temperature of the generated background and the desired temperature, to cover as many different environmental scenarios as possible.

**Figure 4:**
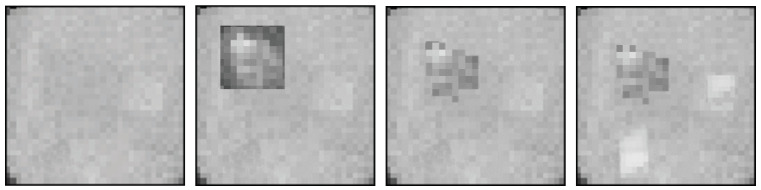
Schematic process of image creation with the generative image creation approach. The sequence is from left to right: generating a background, inserting one or more persons, adjusting the transition and inserting other disturbances.

Occupants and heavy disturbances are inserted as extractions from the remaining 2000 captured and manually labeled images. Thereby, disturbances and humans are tagged with bounding boxes. These boxes are extracted from the images with an additional four surrounding pixels. This value was chosen to obtain two pixels, which is the mean amount of the influenced distance around the object by the radiated heat, and another two pixels to smoothly insert the extracted pixel snippet into the background. After the insertion at a specific selected location, a Gaussian filter is used to blend the snippet seamlessly into the background and to overcome hard transitions at the edges. Due to this approach, real-life poses, like standing, sitting, variations in arm and leg positioning, as well as people’s clothing, are cropped and can be overlaid to the generated background while knowing the exact location. This insertion procedure can be used to insert an arbitrary number of persons with random poses as well as noise, and is summarized in Figure 4.

### 3.3. Algorithm Implementation

The implementation of YOLOv5m [43] and DETR [32] follows the official documentation guidelines. Both include the PyTorch framework. YOLOv5m is trained with the given train routine and adjusted parameters. For the DETR algorithm [32], the officially provided evaluation metrics were used and the train and test procedures were rebuilt in the PyTorch Lighting Framework [44] for ease-of-use and faster training. The DETR Model was implemented from the Hugging Faces implementation (https://huggingface.co/docs/transformers/v4.20.1/en/model_doc/detr#transformers.DetrForObjectDetection (accessed on 2 November 2024)).

## 4. Results

The following sections examine the experimental results. First, the applied performance metrics and the test dataset are explained. Subsequently, multiple tests are performed to evaluate the advantage of the generative model and to test the general robustness as well as the robustness against disturbances, movement and positions, as well as winter clothing and multiple persons.

### 4.1. Model Training

The training of both algorithms took place on a 12 CPU@2.6 GHz (Skylake), 29.3 GB RAM, Windows 10 Pro machine with a 8 GB VRAM Nvidia P4000 GPU. The parameters that differ from the default parameters for YOLOv5 are as follows: batch size: 800, epochs: 200 and number of workers: 2. Within the implementation of the training for DETR, all the aforementioned parameters like batch size and epochs are automatically selected from the PyTorch Lighting Framework. The training utilized, in total, 17,508 generated data from the IIG algorithm, which are based on 3400 captured and manually labeled data from Section 3.2. In total, DETR and YOLOv5m have been trained for 200 epochs. In Table 3, the training results and speed are shown in greater detail. The main difference is the model size with 41 MB for YOLOv5m, which is much lower than the DETR model with 485 MB and has the ability to run on edge computing devices with low memory. Furthermore, the YOLOv5 model took only 2.6 h to train, whereas DETR trained 240 h. With an inference time per image of 1.3 ms for YOLOv5, the algorithm is able to detect objects with the full speed of the sensor, and DETR can process images in 300 ms, which leads to a reduced frame rate.

### 4.2. Performance Metrics

As a suitable evaluation metric, the mean average precision (mAP) is applied to include the correct classification as well as the correct location [45]. To evaluate the location of the bounding box, the average precision (AP) employs the intersection over union (IoU) metric. If the IoU between the predicted bounding box and the ground truth is below a threshold specified by the user, the localization is determined to be incorrect. The mAP is usually calculated in two ways [21,24,25,27,30,32]: Either the mean over all class APs with a single IoU threshold is taken, as is conducted on the PASCAL VOC dataset, or the mAP is calculated for a range of IoU thresholds and then averaged, as is common practice on the MS COCO dataset. For Pascal VOC, a single IoU threshold of >0.5 is defined as being able to calculate the mAP and for MS COCO the mAP is determined by the average of all AP for an IoU between 0.5 and 0.95 with a step size of 0.05, which gives more weight to the exact localization of the object in the image [45]. These two values are used as mAP_0.5_ for the PASCAL VOC value and mAP_>0.5_ for the COCO value in this paper. Since, in the context of this work, the detection of persons primarily plays a major role rather than their exact localization, mAP_0.5_ is applied as the main evaluation metric.

### 4.3. Test Dataset

For evaluation purposes, multiple datasets corresponding to the tests described above were collected and extended with the generative model. The exact involved test datasets are specified in accordance to the respective tests explained in the next sections. As a test scenario, up to five persons are included: four male and one female. The four male participants consist of persons 1 and 2, known from the creation of the training data, as seen in Table 2, along with two new male participants and one female participant. To test the robustness and generalization, a different arrangement of the room objects is established to simulate a new room. The new room configuration is ongoing, and is called Room2.

### 4.4. Generative Model

From the captured 3017 data in Room1, 10% of the data (307) are randomly selected and only used for testing the infrared image generator (IIG). In these test data, the same people and room as in the generated training dataset are present. For Room2 the same four male and one additional female persons were asked to generate in total another 425 labeled test data with a similar procedure as the previous generated training data (see Section 3.1). For training the IIG the 3400 data explained in Section 3.2 are utilized. For augmentation the 2150 training data out of Section 3.1 are included as well as rotated and flipped samples to receive 17,722 training data overall. With the IIG algorithm, 17,508 images are created for training. All training data were captured in Room1. Room2 is only represented in the 425 test data.

In Table 4, a comparison of the mAP values between different trained YOLOv5m algorithms and DETR is represented. The training lasted for 200 epochs for YOLO and DETR. Afterwards, the test datasets for Room1 and Room2 are applied for evaluation. The meaning of the abbreviations in Table 4 are as follows:

- orig: the real measured and manually labeled data;
- gen: data generated with the generative model (IIG);
- aug: data that are augmented by rotating and flipping the measured orig dataset.

Table 4 shows that the test with the highest mAP value is either the generated data (gen) alone or combined with the original measured data (orig). Furthermore, the mAP of Room1 compared to Room2 has only a small decline compared to the augmented or original dataset. Thus, the data generation yields an improvement in precision, a higher robustness to different environments as well as persons and a better generalization regarding the generated data. For the last entry, the gen+orig dateset is used in a way so that the trained model with the generated data (gen) is additionally trained with the measured data (orig). This subsequent training could lead to a decline in generalization, and, therefore, this entry is slightly worse than only the generated data for the newly presented Room2. In summary, both algorithms can localize and detect humans well, with a mAP_0.5_ of 0.975 for DETR and 0.985 for YOLOv5m. A minor reduction in the mAP score is revealed for the new scenario (Room2) with a new person.

It can be seen in Table 4 that YOLOv5m_gen_ and DETR_gen_ have a high mAP after training solely with the generated dataset. These trained models are applied for the evaluation in all the next sections. Due to the long training duration of DETR, it was only trained with the generated data as reference and revealed a slightly worse mAP score as YOLOv5m_gen_ and is therefore not considered for further training on the augmented and original data. The exact training statistics for YOLOv5m and DETR on the generated dataset in Room1 are presented in Section 4.1 and show a substantially faster training time for YOLO and a real-time ability with >8.3 fps as the sensors’ maximum output speed (see Table 1).

### 4.5. General Robustness

As a next step, the general robustness of both algorithms is investigated without disturbing heat-radiating objects but including shelves, chair, desk and walls in the field of view (FoV). For this purpose, five new test datasets were created:

- Reference: test data from Room1 with persons and room as known from the training;
- New Room: Room2 was used with the same four male persons as in the reference;
- New person: a fifth female person was captured in Room1;
- New Room + Person: gathering the new person in Room2;
- Second sensor: nother sensor of the same type—HTPA32 × 32dR2L1.9/0.8HiC[CH]—was applied;
- Different Sensor: similar sensor but with a lower thermal sensitivity that results in a lower signal-to-noise ratio is included: HTPA32x32dR2L2.1/0.8F5.0HiA[Si].

As reference, the 307 data of Room1 were applied, where these data include the same environment (Room1) and four male occupants as examined for the model training that utilizes the 17,508 generated data of the IIG algorithm. The change in environment causes fewer problems for both algorithms than a new person, as the mAP values indicate in Table 5. Furthermore, the YOLOv5 model performs better than DETR for a new person and room, although DETR yielded a higher accuracy on the validation data during training. Both models prove to be robust against change in occupants or new environments with >mAP_0.5_ 0.91 and >mAP_>0.5_ 0.5, respectively.

DETR reveals fewer issues due to the change to an equal type sensor (2. sensor) as far as the detection of a person is concerned, but the YOLOv5 model exhibits a superior ability in localizing persons. This is in contrast to the change to a different sensor model with a poorer signal-to-noise ratio, where YOLOv5m still has a high mAP_0.5_ of 0.884 where DETR has only 0.28. In general, both models show significantly worse detection performance in images with the different sensor. DETR especially lacks robustness to the sensor change and cannot generalize to the poorer image quality. This could be due to the fact that it has adapted too well to the images learned in training (overfitting).

### 4.6. Robustness Against Disturbances

In order to make the algorithm robust against disturbances during the training process, 10,500 images (60%) of the 17,508 generated images contained disturbances. First, two datasets simulating extreme disturbances are created: one with hot and one with cold objects, to determine the respective influence of the temperatures. As disturbance sources, objects that can occur in an office and work environment were selected. For these tests, a dataset is captured with the following hot objects: a kettle, a coffee maker, a cup with a hot beverage, two radiators—one wall heater and one electric radiator—floor heating and two chargers for a laptop and a cell phone, in addition to the previous located standard laptop and monitor heat sources. As for the cold objects, Coolpacks, ice cream, cold drinks and an open fridge and freezer were selected as sources of interference. As reference mAP value, the 425 IR images of Room2 with a new fifth person are applied. Table 6 reveal that the YOLO model is very robust against the mentioned interference sources and exceeds DETR. The influence of heat also seems to be stronger than the influence of cold objects according to this test. This could be due to the fact that a human body also presents a heat source and therefore distinguishing it from interfering heat sources is more challenging. The next two groups of data focus on the specific influence of thermal radiation, such as warm air flow and sun rays through a window that heat the surface. In the first of these three warm air datasets, the hair dryer that simulates the warm air flow is simply directed into the test environment without directly targeting an object. In the second, it directly irradiates the test subject, and in the third it is aimed directly at the sensor. YOLO and DETR both yield a comparable mAP with respect to the reference. Thus, it can be concluded that these disturbances have a minor influence on the detection and localization accuracy.

### 4.7. Robustness of Movement and Position

This section examines the influence of movement, posture and position within the image. For this purpose, in the first test scenario, datasets are created that contain both standing and sitting people at rest and in motion. The datasets were recorded centrally under the sensor except for the datasets named as on the edge, which are images recorded with persons standing and walking on the edge of the field of view of the sensor.

Motion has little influence on the detection of both algorithms, as Table 7 presents. Their mAP_0.5_ values are consistently very high, which shows that they reliably detect the people in the images. Only the exact localization, indicated by the lower mAP_>0.5_ values, seems to be more difficult for YOLOv5 when moving while sitting. DETR shows the worst results for localization when sitting at rest. Detection at the edge of the image is also no issue for either approaches.

### 4.8. Influence of Winter Clothing

Room2 serves as the reference equal to Section 4.6. The test subjects for this evaluation scenario were dressed in spring clothing with both shorts and long pants combined with a t-shirt. For the winter clothing dataset, the test subjects wear a thick winter jacket in addition to long pants in each case. The winter hat dataset adds a hat to the winter outfit. Table 8 shows that a reliable detection of individuals is possible despite them wearing winter clothing. Adding the winter hat significantly decreases the detection. This shows the importance of the large heat radiated from the head for the detection of a person. For this task, the YOLOv5 model is superior to the DETR model.

### 4.9. Multiple Persons

**Figure 5:**
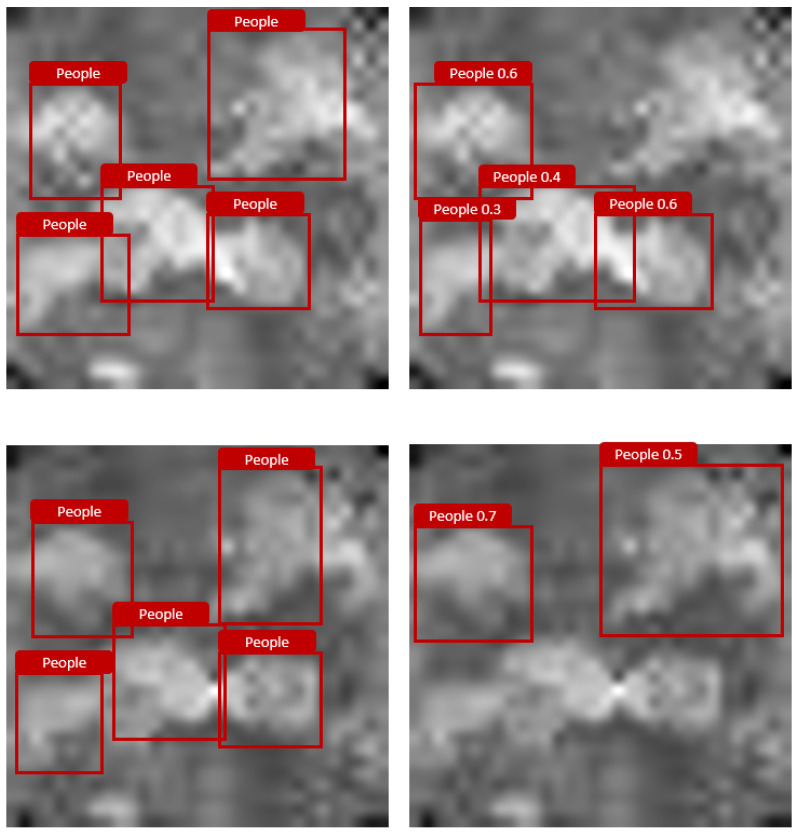
Visualization example of YOLOv5 detection of five people in one image. On the left is the ground truth and on the right is the labels provided by YOLOv5.

As a final evaluation, the influence of multiple persons is examined in a room without objects to obtain sufficient space for up to five people within the 250 × 250 cm field of view of the sensor. In the training dataset, only one or two persons are captured per image. This test determines how well the models have learned the representation or features of people and can generalize to many people in a picture and thus to another situation that is completely unknown to the models. The results of this last evaluation test can be seen in Table 9. YOLOv5 and DETR both showed no problems in detecting one or two persons. For three and four people, YOLO still provides good precision in detection. With five persons in the image, this algorithm encounters noticeable difficulties. In the case of DETR, the trained model already exceeds its limits for the detection of three occupants. Figure 5 presents an example of the detection of two randomly selected images out of the dataset with five persons by YOLO. As the images reveal, these persons that are detected are similar to the ground truth; however, the model is not capable of detecting all the people present in the image.

## 5. Discussion

The goal of this paper was to find and evaluate an algorithm that robustly detects and localizes people with a 32 × 32-pixel thermopile array sensor. Based on a conducted literature research, two single-stage detectors, YOLOv5m and DETR, were selected due to their real-time capability in processing images of the infrared sensor with full speed. YOLO reached 792 fps and is capable of processing the images at full speed. DETR accomplishes 3 fps and is substantially slower, as the given sensors’ output default is 8.3 fps. This observation is similar to the results of the comparison of an object detection algorithm by Zaidi et al. [24]. Given the model size of 41 MB of YOLO, running this algorithm on an edge computing device is more likely than the 485 MB model of DETR. Furthermore, YOLOv5 was trained for 200 epochs in 2.6 h, whereas the best epoch for DETR was 44 and took 53 h. As for training, a set of 3400 images was manually labeled and extended by a novel thermal image generator (IIG) to a total of 17,508 images. This image generator is able to generate an arbitrary number of images from a few labeled data and archives a higher mAP through solely augmenting images with flipping and rotation. This is achieved by creating various backgrounds from the density function of the input data and blending in real cropped and filtered persons and disturbances. The evaluation of the robustness of the two algorithms reveal that after training, YOLOv5m reaches 99% mAP_0.5_ as DETR reaches 98% mAP_0.5_. Both YOLOv5 and DETR provide very accurate models that are well-suited for person detection in thermal images and both have exceptionally high precision and applicability to untrained persons and environments. The change in the sensor to an equivalent type of sensor model is also possible; however, a change to a different sensor model with a poorer signal-to-noise ratio affects the precision and retraining is recommended. In this evaluation, both models achieved over 98% mAP_0.5_ on real data with known individuals and over 95% mAP_0.5_ on real data with untrained people in an unknown environment. However, YOLOv5 was about 1% more accurate than DETR in both categories. YOLO is less affected by untrained interfering heat or cold objects. The mAP_0.5_ of DETR drops for unknown hot objects to 50%, such as in the case of kettles, hot beverages and heaters. A warm air stream and sun presents only a small effect, similar to occupants moving or resting within the field of view and on its edges. A far larger decrease is caused by subjects wearing a winter hat and by adding more persons to the scenery as trained. In summary, it can be stated that YOLOv5m is superior in comparison to DETR.

No clear statement can be obtained about the generalization of these results, since the training of the algorithms and the creation and selection of the trainings as well as test data may favor one of the two models. In addition, the choice of methods was made from a very narrow set, so there is a possibility of the existence of a much more suitable model that is not considered in this work, like Swin Transformer [34]. Also, the focus on mAP might add a bias to the results of this study or might not represent them comprehensively enough. Therefore, these algorithims cannot be assumed to have found a generally optimal option. In further work, the classification can be extended to multiple classes like sitting, standing or to lying, and disturbances could be labeled. The results of the localization might also be used to create trajectories and obtain a better understanding of movement inside the field of view. Despite the limitations, it can be assumed that the created YOLOv5m model is a detector that delivers very good precision and fulfills the requirements for this work.

## 6. Conclusions

This paper evaluates two single-stage detectors, YOLOv5m and DETR, as person detection and localization approaches in 32 × 32-pixel thermal images. By analyzing multiple scenarios and disturbances, both algorithms present a good precision of 99% for YOLOv5m and 98% for DETR, and can therefore both be applied for tasks. Likewise, both algorithms have a high tolerance to disturbing sources based on the training dataset created by the generative model. YOLOv5 has a much faster inference time and a smaller model, so it is more suitable for running on edge computing devices. Furthermore, YOLOv5m is more robust against multiple disturbances.

## Figures and Tables

**Table 1:** Specifications of the thermopile array sensor HTPA32 × 32dR2L1.9/0.8HiC[Ch] from Heimann Sensor GmbH.

Parameter	Value
clock frequency (sensor)	5 MHz ± 3 MHz
ambient temperature range	−20–85 °C
object temperature range	−2–1000 °C
frame rate	2–27 (default 8.3) fps
thermal sensitivity (NETD)	173 mK@1 Hz
field of view FoV	96 × 96 deg
resolution	32 × 32 pixel

**Table 2:** Height and hair type of four male participants used in three scenarios of the experiment.

Participant	Height in cm	Hair Type
1	184	long
2	166	few, short
3	178	short
4	181	short

**Table 3:** Trainings speed for YOLOv5 and DETR.

	YOLOv5m	DETR
# images in training dataset	17,508	17,508
training duration	∼2.6 h	∼240 h
training epochs	200	200
duration/epoch	∼47 s	∼1.2 h
best result epoch	83	44
validation accuracy for best epoch	0.976 mAP_0.5_	0.986 mAP_0.5_
inference time per image	1.3 ms (769 fps)	300 ms (3 fps)
model size	41 MB	485 MB

**Table 4:** Evaluation results for the YOLOv5m models and different training datasets. Bold marked values represent the best values per column. The mAP values are calculated based on the 307 data from Room1 and 425 data from Room2.

Model	# Training Data	Room1		Room2	
		**mAP_0.5_**	**mAP_>0.5_**	**mAP_0.5_**	**mAP_>0.5_**
YOLOv5m_orig_	2150	0.557	0.263	0.0724	0.116
YOLOv5m_aug_	17,722	0.941	0.493	0.895	0.414
YOLOv5m_gen_	17,508	0.995	0.626	**0.985**	**0.616**
YOLOv5m_gen+orig_	19,658	**0.998**	**0.632**	0.945	0.483
DETR_gen_	17,508	0.989	0.601	0.975	0.601

**Table 5:** General robustness of the two algorithms. Comparison of test data similar to the training, with a new person and in a new environment as well as the change to an equal sensor type and a different sensor type with poorer signal-to-noise ratio and the same resolution. Bold numbers indicate the better values.

Model	Reference		New Room		New Person	
	mAP_0.5_	mAP_>0.5_	mAP_0.5_	mAP_>0.5_	mAP_0.5_	mAP_>0.5_
YOLOv5m	**0.995**	**0.626**	**0.985**	**0.616**	**0.980**	0.532
DETR	0.975	0.601	0.975	0.601	0.950	**0.542**
# images:	307		425		257	
	New Room + Person		2. Sensor		Different Sensor	
	mAP_0.5_	mAP_>0.5_	mAP_0.5_	mAP_>0.5_	mAP_0.5_	mAP_>0.5_
YOLOv5m	**0.955**	0.502	0.968	**0.595**	**0.884**	**0.449**
DETR	0.915	**0.511**	**0.982**	0.545	0.280	0.134
# images:	263		137		124	

**Table 6:** Influence of heat and cold on the two algorithms. Bold numbers indicate the better values. Bold numbers indicate the better values.

Model	Reference		Heat		Cold		Sun	
	mAP_0.5_	mAP_>0.5_	mAP_0.5_	mAP_>0.5_	mAP_0.5_	mAP_>0.5_	mAP_0.5_	mAP_>0.5_
YOLOv5m	**0.985**	**0.616**	**0.946**	**0.563**	**0.980**	**0.566**	**0.994**	**0.592**
DETR	0.975	0.601	0.509	0.310	0.962	0.546	0.985	0.586
# images:	425		879		173		157	
	Warm Air							
	In The Room		On Person		On Sensor			
	mAP_0.5_	mAP_>0.5_	mAP_0.5_	mAP_>0.5_	mAP_0.5_	mAP_>0.5_		
YOLOv5m	**0.990**	**0.555**	**0.995**	**0.623**	**0.995**	**0.683**		
DETR	0.987	0.506	0.988	0.564	0.985	0.538		
# images:	225		168		137			

**Table 7:** Influence of movement and rest on the two algorithms. Bold numbers indicate the better values.

Model	Movement							
	Walking		Standing		Sitting		On The Edge	
	mAP_0.5_	mAP_>0.5_	mAP_0.5_	mAP_>0.5_	mAP_0.5_	mAP_>0.5_	mAP_0.5_	mAP_>0.5_
YOLOv5m	**0.992**	**0.651**	**0.995**	**0.607**	**0.995**	0.507	**0.988**	**0.576**
DETR	0.989	0.579	0.983	0.567	0.989	**0.534**	0.939	0.542
# images:	207		164		179		248	
Rest								
	Standing		Sitting		On The Edge			
	mAP_0.5_	mAP_>0.5_	mAP_0.5_	mAP_>0.5_	mAP_0.5_	mAP_>0.5_		
YOLOv5m	**0.995**	**0.637**	**0.995**	**0.602**	**0.994**	0.513		
DETR	0.988	0.614	0.987	0.476	0.973	**0.552**		
# images:	258		143		157			

**Table 8:** Influence of winter clothing and a winter hat. Bold numbers indicate the better values.

Model	Reference		Winter Cloth		Winter Hat	
	**mAP_0.5_**	**mAP_>0.5_**	**mAP_0.5_**	**mAP_>0.5_**	**mAP_0.5_**	**mAP_>0.5_**
YOLOv5m	**0.985**	**0.616**	**0.913**	**0.364**	**0.515**	**0.142**
DETR	0.975	0.601	0.881	0.332	0.267	0.104
# images:	425		151		51	

**Table 9:** Influence of multiple people on trained models with up to two persons. Bold numbers indicate the better values.

Model	1 Person		2 Persons		3 Persons	
	mAP_0.5_	mAP_>0.5_	mAP_0.5_	mAP_>0.5_	mAP_0.5_	mAP_>0.5_
YOLOv5m	**0.995**	**0.588**	**0.991**	0.623	**0.835**	**0.356**
DETR	0.977	0.554	0.887	**0.670**	0.483	0.132
# images:	77		80		114	
	4 Persons		5 Persons			
	mAP_0.5_	mAP_>0.5_	mAP_0.5_	mAP_>0.5_		
YOLOv5m	**0.799**	**0.282**	**0.539**	**0.148**		
DETR	0.386	0.142	0.298	0.117		
# images:	107		103			

## Data Availability

The novel generative image generator (IIG) algorithm, as well as the original data presented in this paper, are openly available on Github: https://github.com/KlirS/Multi-Person-Localization-based-on-a-Thermopile-Array-Sensor-with-a-Generative-Data-Model (accessed on 2 November 2024).

## References

[1] Klir S., Fathia R., Benkner S., Babilin S., Khanh T.Q. Preference Lighting Model: Generalization of lighting preferences for individual users. Proceedings of the 2021 Joint Conference—11th International Conference on Energy Efficiency in Domestic Appliances and Lighting & 17th International Symposium on the Science and Technology of Lighting (EEDAL/LS:17).

[2] Fan X., Zhang H., Leung C., Shen Z. Robust unobtrusive fall detection using infrared array sensors. Proceedings of the 2017 IEEE International Conference on Multisensor Fusion and Integration for Intelligent Systems (MFI).

[3] Hayashida A., Moshnyaga V., Hashimoto K. The use of thermal IR array sensor for indoor fall detection. Proceedings of the 2017 IEEE International Conference on Systems, Man, and Cybernetics (SMC).

[4] Morawski I., Lie W.N., Chiang J.C. Action Prediction Using Extremely Low-Resolution Thermopile Sensor Array for Elderly Monitoring. Proceedings of the 2021 IEEE International Conference on Image Processing (ICIP).

[5] Shetty A.D., Disha, Shubha B., Suryanarayana K. Detection and tracking of a human using the infrared thermopile array sensor—‘Grid-EYE’. Proceedings of the 2017 International Conference on Intelligent Computing, Instrumentation and Control Technologies (ICICICT).

[6] Tariq O.B., Lazarescu M.T., Lavagno L. (2021). Neural Networks for Indoor Person Tracking with Infrared Sensors. IEEE Sens. Lett..

[7] Corsi C. (2010). History highlights and future trends of infrared sensors. J. Mod. Opt..

[8] Gu N., Yang B., Li T. High-resolution Thermopile Array Sensor-based System for Human Detection and Tracking in Indoor Environment. Proceedings of the 2020 15th IEEE Conference on Industrial Electronics and Applications (ICIEA).

[9] Chen Z., Wang Y., Liu H. (2018). Unobtrusive sensor-based occupancy facing direction detection and tracking using advanced machine learning algorithms. IEEE Sens. J..

[10] Gochoo M., Tan T.-H., Batjargal T., Seredin O., Huang S.-C. Device-Free Non-Privacy Invasive Indoor Human Posture Recognition Using Low-Resolution Infrared Sensor-Based Wireless Sensor Networks and DCNN. Proceedings of the 2018 IEEE International Conference on Systems, Man, and Cybernetics (SMC).

[11] Tateno S., Meng F., Qian R., Hachiya Y. (2020). Privacy-Preserved Fall Detection Method with Three-Dimensional Convolutional Neural Network Using Low-Resolution Infrared Array Sensor. Sensors.

[12] Petrova G., Spasov G., Iliev I. A Review on Applications of Low-resolution IR Array Sensors in Ambient-Assisted Living. Proceedings of the 2021 XXX International Scientific Conference Electronics (ET).

[13] Parnin S., Rahman M.M. (2017). Human location estimation using thermopile array sensor. IOP Conf. Ser. Mater. Sci. Eng..

[14] Shubha B., Shastrimath V.V.D. (2022). Real-Time Occupancy Detection System Using Low-Resolution Thermopile Array Sensor for Indoor Environment. IEEE Access.

[15] Honorato J.L., Spiniak I., Torres-Torriti M. Human detection using thermopiles. Proceedings of the 2008 IEEE Latin American Robotic Symposium.

[16] Trofimova A.A., Masciadri A., Veronese F., Salice F. (2017). Indoor Human Detection Based on Thermal Array Sensor Data and Adaptive Background Estimation. J. Comput. Commun..

[17] Perra C., Kumar A., Losito M., Pirino P., Moradpour M., Gatto G. (2021). Monitoring indoor people presence in buildings using low-cost infrared sensor array in doorways. Sensors.

[18] Vandersteegen M., Reusen W., Beeck K.V., Goedemé T. (2022). Person Detection Using an Ultra Low-resolution Thermal Imager on a Low-cost MCU. Image and Vision Computing. IVCNZ 2022. Lecture Notes in Computer Science.

[19] Uijlings J.R.R., Sande K.E.A.V.D., Gevers T., Smeulders A.W.M. (2013). Selective search for object recognition. Int. J. Comput. Vis..

[20] Zou Z., Shi Z., Guo Y., Ye J. (2023). Object Detection in 20 Years: A Survey. Proc. IEEE.

[21] Wu X., Sahoo D., Hoi S.C.H. (2020). Recent advances in deep learning for object detection. Neurocomputing.

[22] Girshick R., Donahue J., Darrell T., Malik J. (2016). Region-Based Convolutional Networks for Accurate Object Detection and Segmentation. IEEE Trans. Pattern Anal. Mach. Intell..

[23] Liu L., Ouyang W., Wang X., Fieguth P., Chen J., Liu X., Pietikäinen M. (2020). Deep Learning for Generic Object Detection: A Survey. Int. J. Comput. Vis..

[24] Zaidi S.S.A., Ansari M.S., Aslam A., Kanwal N., Asghar M., Lee B. (2022). A survey of modern deep learning based object detection models. Digit. Signal Process. A Rev. J..

[25] Girshick R. (2015). Fast R-CNN. Proc. IEEE Int. Conf. Comput. Vis..

[26] Dai J., Li Y., He K., Sun J. (2016). R-FCN: Object detection via region-based fully convolutional networks. Adv. Neural Inf. Process. Syst..

[27] Ren S., He K., Girshick R., Sun J. (2017). Faster R-CNN: Towards Real-Time Object Detection with Region Proposal Networks. IEEE Trans. Pattern Anal. Mach. Intell..

[28] Lin T.Y., Dollár P., Girshick R., He K., Hariharan B., Belongie S. Feature pyramid networks for object detection. Proceedings of the 30th IEEE Conference Computer Vision Pattern Recognition, CVPR 2017.

[29] He K., Gkioxari G., Dollár P., Girshick R. (2020). Mask R-CNN. IEEE Trans. Pattern Anal. Mach. Intell..

[30] Redmon J., Divvala S., Girshick R., Farhadi A. (2016). You only look once: Unified, real-time object detection. Proc. IEEE Comput. Soc. Conf. Comput. Vis. Pattern Recognit..

[31] Wang C.-Y., Bochkovskiy A., Liao H.-Y.M. YOLOv7: Trainable bag-of-freebies sets new state-of-the-art for real-time object detectors. Proceedings of the IEEE/CVF Conference on Computer Vision and Pattern Recognition.

[32] Carion N., Massa F., Synnaeve G., Usunier N., Kirillov A., Zagoruyko S. (2020). End-to-End Object Detection with Transformers. European Conference on Computer Vision.

[33] Lin T.-Y., Maire M., Belongie S., Bourdev L., Girshick R., Hays J., Perona P., Ramanan D., Zitnick C.L., Dollár P. (2015). Microsoft COCO: Common Objects in Context. arXiv.

[34] Liu Z., Lin Y., Cao Y., Hu H., Wei Y., Zhang Z., Lin S., Guo B. Swin Transformer: Hierarchical Vision Transformer using Shifted Windows. Proceedings of the IEEE/CVF International Conference on Computer Vision.

[35] Song H., Sun D., Chun S., Jampani V., Han D., Heo B., Kim W., Yang M.H. (2022). An Extendable, Efficient and Effective Transformer-based Object Detector. arXiv.

[36] Anyanwu G.O., Nwakanma C.I., Putri A.R., Lee J.M., Kim D.S., Kim J., Hwang G. Thermal Array Sensor Resolution-Aware Activity Recognition using Convolutional Neural Network. Proceedings of the 2022 International Conference on Artificial Intelligence in Information and Communication (ICAIIC).

[37] Chen W.H., Ma H.P. A fall detection system based on infrared array sensors with tracking capability for the elderly at home. Proceedings of the 2015 17th International Conference on E-Health Networking, Application & Services (HealthCom).

[38] Mashiyama S., Hong J., Ohtsuki T. Activity recognition using low resolution infrared array sensor. Proceedings of the 2015 IEEE International Conference on Communications (ICC).

[39] Muthukumar K.A., Bouazizi M., Ohtsuki T. (2021). A Novel Hybrid Deep Learning Model for Activity Detection Using Wide-Angle Low-Resolution Infrared Array Sensor. IEEE Access.

[40] Han K., Wang Y., Chen H., Chen X., Guo J., Liu Z., Tang Y., Xiao A., Xu C., Xu Y. (2022). A Survey on Vision Transformer. IEEE Trans. Pattern Anal. Mach. Intell..

[41] Ahmad M., Ahmed I., Ullah K., Khan I., Khattak A., Adnan A. (2019). Person detection from overhead view: A survey. Int. J. Adv. Comput. Sci. Appl..

[42] Taramasco C., Rodenas T., Martinez F., Fuentes P., Munoz R., Olivares R., De Albuquerque V.H., Demongeot J. (2018). A novel monitoring system for fall detection in older people. IEEE Access.

[43] Jocher G., Chaurasia A., Stoken A., Borovec J., Kwon Y., Michael K., Fang J., Yifu Z., Wong C., Montes D. (2022). ultralytics/yolov5: V7.0-YOLOv5 SOTA Realtime Instance Segmentation (v7.0).

[44] Falcon W., The PyTorch Lightning Team (2023). PyTorch Lightning (2.0.8).

[45] Padilla R., Netto S.L., Silva E.A.B.D. A Survey on Performance Metrics for Object-Detection Algorithms. Proceedings of the 2020 International Conference on Systems, Signals and Image Processing (IWSSIP).

